# High-Salt Diet Has a Certain Impact on Protein Digestion and Gut Microbiota: A Sequencing and Proteome Combined Study

**DOI:** 10.3389/fmicb.2017.01838

**Published:** 2017-09-21

**Authors:** Chao Wang, Zixin Huang, Kequan Yu, Ruiling Ding, Keping Ye, Chen Dai, Xinglian Xu, Guanghong Zhou, Chunbao Li

**Affiliations:** ^1^Jiangsu Collaborative Innovation Center of Meat Production and Processing, Quality and Safety Control Nanjing, China; ^2^Key Laboratory of Meat Processing and Quality Control, Ministry of Education Nanjing, China; ^3^Key Laboratory of Meat Products Processing, Ministry of Agriculture Nanjing, China; ^4^College of Food Science, Nanjing Agricultural University Nanjing, China

**Keywords:** high salt, gut microbiota, feces, proteome, 16S rRNA sequencing

## Abstract

High-salt diet has been considered to cause health problems, but it is still less known how high-salt diet affects gut microbiota, protein digestion, and passage in the digestive tract. In this study, C57BL/6J mice were fed low- or high-salt diets (0.25 vs. 3.15% NaCl) for 8 weeks, and then gut contents and feces were collected. Fecal microbiota was identified by sequencing the V4 region of 16S ribosomal RNA gene. Proteins and digested products of duodenal, jejunal, cecal, and colonic contents were identified by LC-MS-MS. The results indicated that the high-salt diet increased Firmicutes/Bacteroidetes ratio, the abundances of genera Lachnospiraceae and *Ruminococcus* (*P* < 0.05), but decreased the abundance of *Lactobacillus* (*P* < 0.05). LC-MS-MS revealed a dynamic change of proteins from the diet, host, and gut microbiota alongside the digestive tract. For dietary proteins, high-salt diet seemed not influence its protein digestion and absorption. For host proteins, 20 proteins of lower abundance were identified in the high-salt diet group in duodenal contents, which were involved in digestive enzymes and pancreatic secretion. However, no significant differentially expressed proteins were detected in jejunal, cecal, and colonic contents. For bacterial proteins, proteins secreted by gut microbiota were involved in energy metabolism, sodium transport, and protein folding. Five proteins (cytidylate kinase, trigger factor, 6-phosphogluconate dehydrogenase, transporter, and undecaprenyl-diphosphatase) had a higher abundance in the high-salt diet group than those in the low-salt group, while two proteins (acetylglutamate kinase and PBSX phage manganese-containing catalase) were over-expressed in the low-salt diet group than in the high-salt group. Consequently, high-salt diet may alter the composition of gut microbiota and has a certain impact on protein digestion.

## Introduction

Epidemiological evidence suggested that the intake of salt, especially sodium chloride, may increase the risk of non-communicable diseases such as hypertension, stroke, and other cardiovascular diseases (Meneton et al., 2005; He and MacGregor, 2009; Strazzullo et al., 2009; Mugavero et al., 2014; Millen et al., 2016). Besides, an increased incidence to cardiovascular disease was associated with excessive consumption of processed meats, partially due to increased salt intake (Micha et al., 2012). Animal studies declared that high-salt diet was regarded to have an adverse effect on gut health, by causing aggravation of tissue inflammation and autoimmune diseases (Yi et al., 2015). Moreover, high-salt diet may cause epithelial proliferation, apoptosis, and altered cellular types (Xiao et al., 2005). Gut microbiota plays an important role in nutritional and immunological processes (Putignani et al., 2014), and changes in the composition of the gut microbiota may have an impact on human health (Clemente et al., 2012). However, few data are available on the effect of high-salt diet on gut microbiota. A study indicated that high-salt diet increased the vulnerability to colitis, which was independent of Th17 cells pathway but associated with shifts in gut microbiota. Specifically, Lachnospiraceae and *Ruminococcus* operational taxonomic units (OTUs) were increased whereas *Lactobacillus* and *Anaerostipes* OTUs were decreased with a loss in diversity (Miranda et al., 2016). A significant decrease in the genus *Providencia* and *Proteus* was detected in feces, indicating that high-salt diet played an important role in shaping gut microbiota during the feeding of sows (Trawńska et al., 2013). However, a salt-tolerant gene which belongs to *Bacteroides* has been proven to avoid the influence of high salt (Culligan et al., 2014), which seems to explain that high-salt diet may induce high proliferation of certain gut microbiota.

In addition, high salt intake may also affect protein expression in different organs, e.g., kidney, heart, and blood vessels, causing an upregulation of fatty acid oxidation enzymes including transketolase, electron-transferring-flavoprotein dehydrogenase, and acyl-coenzyme (Obih and Oyekan, 2014). However, the influence of high salt intake on protein digestion in the digestive tract would become more complex because proteins excreted from the host and gut microbiota may also be degraded by digestive enzymes and gut microbiota. Dietary protein composition had an impact on protein digestion in gastrointestinal tract (Wen et al., 2015). Excessive dietary protein intake would stimulate the growth of *Clostridium perfringens*, and reduce beneficial bifidobacteria in feces (Rist et al., 2013). A high-fat diet would increase the expression of lipid-metabolic enzymes (Park et al., 2004), and glutamate metabolic pathways (Daniel et al., 2014). However, less has been known about alterations in gut microbiota, protein digestion, and absorption in response to a high-salt diet.

The purpose of this study was to demonstrate how a high-salt diet affected the composition of gut microbiota, and the extent of protein digestion and absorption. A 16S rRNA sequencing methodology was applied to monitor the diversity of gut micobiota, and MS-based proteomics was used to characterize the proteome of dietary, host, and bacterial proteins in response to a high-salt diet.

## Materials and Methods

### Animals and Diets

The experimental protocol was approved by the Ethical Committee of Experimental Animal Center of Nanjing Agricultural University. Ten male C57BL/6J mice were obtained from Nanjing Biomedical Research Institute of Nanjing University and housed in a specific pathogen-free animal center (SYXK < Jiangsu > 2011-0037). After 2 weeks adaptation, the mice were randomly assigned to a high-salt diet group or a low-salt diet group. Mice were fed the nutritionally balanced semi-synthetic diets for 8 weeks with *ad libitum* to diets and water in a humidity (60 ± 10%) and temperature (20.0 ± 0.5°C) controlled room with a 12 h light–dark cycle.

Low- and high-salt diets (0.25 vs. 3.15% NaCl) were prepared according to the AIN-93G diet to satisfy the nutritional requirements for growing mice that differed in salt content. Casein was obtained from Shanghai Ruian Bio Technologies Co., Ltd. The diets comprise of protein (20%), cornstarch (39.75%), dextrinized cornstarch (13.2%), sucrose (10%), soybean oil (7%), fiber (5%), mineral mix (3.5%), vitamin mix (1%), cystine (0.3%), choline bitartrate (0.25%), and *tert*-butylhydroquinone (0.0014%).

### Sample Collection

After 8-week feeding, all mice were decapitated, and blood was collected in Eppendorf tubes. The tubes stand at room temperature for 45 min and then were centrifuged at 12,000 × *g* for 30 min to pellet the blood cells. Serum samples were collected and stored at -80°C until analyses. The contents in duodenum, jejunum, cecum and colon, and feces were frozen in liquid nitrogen, and stored at -80°C for further analyses. Intestinal contents were used for proteomic analysis. Feces were used for 16S rRNA sequencing.

### Serum Inflammatory Cytokines

The serum inflammatory cytokines, IL-1β, IL-6, IL-10, IL-17A, IFN-γ, and TNF-α, were determined using a commercial Bio-Plex kit (5827, Bio-Rad Laboratories, Inc.) according to the manufacture’s protocols.

### 16S rRNA Sequencing

#### DNA Extraction and PCR Amplification

Microbial DNA was extracted from fecal samples using the QIAamp DNA Stool Mini Kit (No. 51504, Qiagen, Germany) according to the manufacturer’s protocols. The V4 region of the bacterial 16S ribosomal RNA gene were amplified by using primers 515F 5′-barcode-GTGCCAGCMGCCGCGG-3′ and 806R 5′-GGACTACHVGG GTWTCTAAT-3′, where barcode is an eight-basic sequence unique to each sample. After PCR reactions, amplicons were extracted from 2% agarose gel and purified using the AxyPrep DNA gel extraction kit (Axygen Biosciences, Union City, CA, United States) according to the manufacturer’s instructions and quantified using QuantiFluor^TM^-ST (Promega, United States.).

#### Library Construction and Sequencing

Purified PCR products were quantified by Qubit^®^3.0 (Life Technologies) and every 24 amplicons with different barcodes were mixed. The pooled DNA product was used to construct Illumina Pair-End library following Illumina’s genomic DNA library preparation procedure. Then the amplicon library was paired-end sequenced (2 × 250) on an Illumina MiSeq platform (Shanghai Biozeron Co., Ltd.) according to the standard protocols.

#### Processing of Sequencing Data

Raw fastq files were demultiplexed, quality-filtered using QIIME (version 1.9.0)^1^ with the following criteria: (i) The 250 bp reads were truncated at any site receiving an average quality score <20 over a 10 bp sliding window, and the truncated reads shorter than 50 bp were discarded. (ii) Reads were matched, and two nucleotide mismatching was allowed, but reads containing ambiguous characters were removed. (iii) Sequences that overlap longer than 10 bp were assembled according to their overlap sequencing. Reads that could not be assembled were discarded. OTUs were clustered with 97% similarity cutoff using UPARSE (version 7.1)^2^ and chimeric sequences were identified and removed using UCHIME. The phylogenetic affiliation of each 16S rRNA gene sequence was analyzed by RDP Classifier^3^ against the silva (SSU123) 16S rRNA data basic using confidence threshold of 70% (Amato et al., 2013).

#### Microbial Diversity Analyses

The rarefaction analysis based on Mothur v.1.21.1 (Schloss et al., 2009) was conducted to reveal the diversity indices, including the Chao, ACE, and Shannon diversity indices. The beta diversity analysis was performed using UniFrac (Lozupone et al., 2011) to compare the results of the principal component analysis by using the community ecology package in R (Vegan 2.0 package). Venn diagrams and clustering analysis were performed using Vegan packages in R^4^.

#### LEfSe Analysis

LEfSe (linear discriminant analysis effect size) analysis was performed^5^ to find biomarkers for highly dimensional fecal bacteria (Segata et al., 2011). Kruskal–Wallis sum-rank test was applied to identify the difference between classes followed by LDA analysis to detect the effect size of each differentially abundant taxon. The parameters for LEfSe analysis were set as follows: (1) alpha value for the factorial Kruskal–Wallis test among classes was less than 0.05; (2) alpha value for the pairwise Wilcoxon test among subclasses was less than 0.05; (3) the threshold on the logarithmic LDA score for discriminative features was less than 2.0; (4) the strategy for multi-class analysis was set as all-against-all (Zhu et al., 2015).

### Proteome Analysis

#### Protein Extraction and Digestion

Duodenal, jejunal, cecal, and colonic contents were diluted (1:10, w/v) in RIPA lysis buffer (Beyotime, P0013B), which contained protease inhibitor (1%, v/v, Sigma, P8340) and phosphatase inhibitor (1%, v/v, Sigma, P2850). The samples were homogenized on ice at 8,500 rpm for 60 s each, and the step was repeated three times. Then the samples were centrifuged at 14,000 × *g* at 4°C for 15 min and the supernatant was carefully collected. The centrifugation was repeated once. Protein concentrations were quantified by a BCA protein assay (Bio-Rad, United States), and stored at -80°C before use.

Each sample (200 μg protein) was treated with 200 μL denaturation buffer (8 M urea, 50 mM Tris–HCl, pH 8.0) and 5 μL of DTT (1 M) for 60 min at 60°C, then reduced with 200 μL denaturation buffer (8 M urea, 50 mM Tris–HCl, pH 8.0) and alkylated with 20 μL of iodoacetamide (0.5 M) in dark for 45 min at room temperature. Proteins were diluted with 200 μL of ammonium bicarbonate (50 mM, pH 7.8) and digested with 20 μL of trypsin (0.1 mg/mL) for 16 h at 37°C. Digestion was stopped by adding formic acid (final concentration 0.2%). The resulting mixture was desalted using C18 ZipTip pipette tips and resuspended in 0.1% formic acid. The peptides concentration was determined at 280 nm by using a UV spectrophotometer. The same bulk concentration level was chosen to satisfy the needs for comparisons at the same level. Finally, the samples were dried by vacuum centrifugation for preparation.

#### LC-MS/MS

The peptide samples (1.5 μg) were isolated by reverse-phase liquid chromatography using a nano LC system (DIO-NEX, Thermo Fisher Scientific). After separation, the peptides were analyzed by tandem mass spectrometry (Thermo Fisher Scientific) on a LTQ Orbitrap mass spectrometer according to previous protocols (Hauck et al., 2010). In brief, peptide samples were acidified in 0.1% FA and then loaded onto a nano LC system with C18 column (Acclaim PepMap100, 75 μm × 2 cm, 3 μm, 100 Å, Thermo Fisher Scientific). The loading buffer (2% acetonitrile, 0.1% FA in HPLC-grade water) was run at the flow rate of 4 μL/min. Then, peptide samples were eluted and isolated on an analytical column (Acclaim PepMap RSLC, C18, 75 μm × 15 cm, 3 μm, 100 Å, Thermo Fisher Scientific) with a gradient from 3 to 55% of buffer B (80% acetonitrile, 0.1% FA in HPLC-grade water) for about 112 min at 300 nL/min. After elution, the rest of peptides were eluted by a gradient from 55 to 98% of buffer B for a short time. The selected peptides were analyzed by the LTQ Orbitrap XL. The intense peptide ions were prepared for analysis. The normalized collison energy for CID was set to a value of 35 and the high quality fragments were detected with normal resolution in the linear ion trap. The background signal with the mass of 445.120020 was used as lock mass. Every ion selected for fragmentation was excluded for 60 s by dynamic exclusion (Shi et al., 2016).

#### Database Searches and Bioinformatics Analysis

The raw LC–MS/MS spectra data from samples were matched with MaxQuant search and filtered in the Perseus software package v 1.5.8.3 (Max Planck Institute of Biochemistry). The parameters for searching were set as follows: main search ppm: 4.5; missed cleavage: 2; MS/MS tolerance ppm: 20; de-isotopic: TRUE; enzyme: trypsin, searching database: *Mus musculus, Bos taurus*, and a collection of 81 different bacterial database which were downloaded from the Uniprot; fixed modification: carbamidomethyl (cys); variable modification: oxidation (met), acetyl (protein N-term); decoy database pattern: reverse; label free quantification (LFQ): TRUE; LFQ min ratio count: 1; match between runs: 2 min; peptide FDR: 0.01; and protein FDR: 0.01. Student’s *t*-test was applied for comparisons of quantitative data. After potential contaminants were removed, each protein or protein groups was required to have valid reporter intensity in at least two biological replicates in each experimental group. Proteins were also removed that could not be annotated to gene IDs. Uniprot accession numbers were obtained by using the ID mapping function^6^. Protein sequences were matched via batch retrieval at the protein information resource website^7^. OmicsBean, the multi-omics data analysis tool, was adopted to analyze the selected proteome data^8^, in which distributions in biological functions, subcellular locations, and molecular functions were allocated to each protein which was based on Gene Ontology (GO) categories. The Kyoto Encyclopedia of Genes and Genomes (KEGG) pathway analysis was conducted to enrich the high-level functions in the defined biological systems (Sun et al., 2015). Protein–protein interaction analysis was performed while maintaining the original interacting complex.

## Results

### Growth Performance and Physiological Responses

**FIGURE 1:**
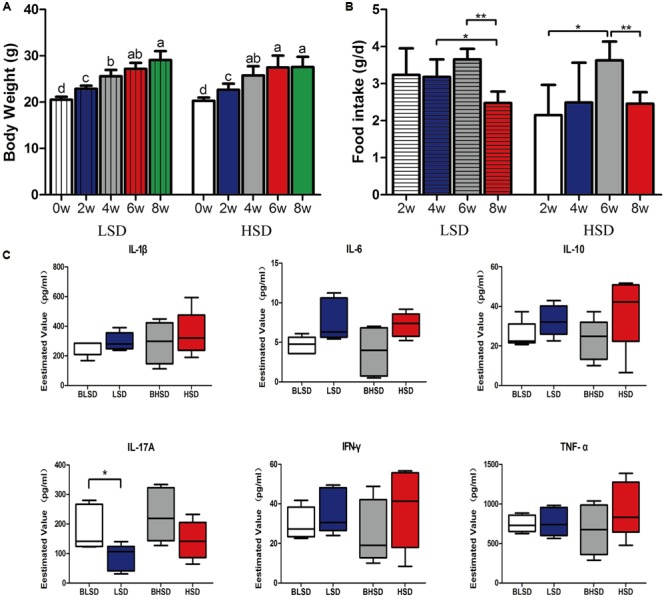
Growth performance and physiological responses of mice. **(A)** The changes of body weight. Each column represents one group. **(B)** The changes of feed intake. **(C)** Box-plots of serum inflammatory factors. The asterisk (^∗^) indicates significant difference between two groups (*P* < 0.05, two sample *t*-test). ^∗^*P* < 0.05; ^∗∗^*P* < 0.01. Note: The data were analyzed by one-way analysis of variance and means were compared by two sample *t*-test. BLSD, low-salt diet group before feeding; LSD, low-salt diet group after feeding; BHSD, high-salt diet group before feeding; HSD, high-salt diet group after feeding.

There was no significant difference (*P* > 0.05; **Figure 1A**) in initial body weight between the low- and the high-salt diet groups. After 8 weeks feeding, the body weight increased greatly (*P* < 0.05), but none of final body weight, weight gain, and feed intake was observed significantly different between the two diet groups (*P* > 0.05). For the low-salt diet group, feed intake kept constant within the first 6 weeks but decreased afterward (*P* < 0.05; **Figure 1B**). For the high-salt diet group, feed intake increased within the first 6 weeks and then decreased afterward (*P* < 0.05; **Figure 1B**). And thus, the high-salt diet seemed not to affect animal growth performance. In addition, none of inflammatory cytokines, e.g., IL-1β, IL-6, IL-10, IL-17A, IFN-γ, and TNF-α were shown significantly different between the low- and the high-salt diet groups (*P* > 0.05; **Figure 1C**). However, IL-17A showed significant difference between two feeding time points in the low-salt diet group (*P* < 0.05; **Figure 1C**).

### Gut Microbiota

#### Richness and Diversity Analyses of Fecal Samples

Four fecal samples from two mice were excluded for statistical analyses because they showed much fewer reads than the other samples. In total, 476,640 bacterial 16S rRNA usable raw reads were detected from all 16 samples with an average of 29,790 reads per sample. On the OTU level, 4,465 OTUs were matched, with an average of 279 ± 44 OTUs per sample. There was no significant difference (*P* > 0.05) between any two groups in Good’s coverage index, ACE, Chao, Shannon index, and Simpson index (Supplementary Table S1). Moreover, the rarefaction result based on OTU level showed no significant difference between samples (**Supplementary Figure S1**). These results indicate that all the samples showed a great similarity in general microbial diversity.

#### Structure of Fecal Microbiota

**FIGURE 2:**
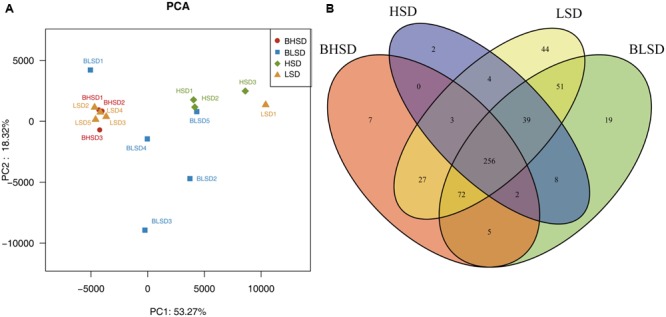
General information on gut microbiota. **(A)** Principal component analysis of fecal microbiota on the OTU level. Each point represents one sample. **(B)** Venn diagram of fecal microbiota on the OTU level. Each ellipse represents one group. Note: The numbers of animals for high-salt diet and low-salt diet groups were 3 and 5, respectively. BHSD, high-salt diet group before feeding; BLSD, basic low-salt diet group before feeding; HSD, high-salt diet group after feeding; LSD, low-salt diet group after feeding.

Multivariate analyses were performed to compare the overall composition of fecal microbiota among all samples on the OTU level. Principal component analysis was applied to visualize the differences in fecal bacteria composition between diet groups before and after feeding. The first two components accounted for 71.59% of the total variation (**Figure 2A**). PC1 explained the variations mainly derived from both diet and feeding time effects, while PC2 explained the intrinsic variations derived from individuals in the low-salt diet group before feeding (BLSD group). For the HSD groups, there was a high variation between groups mainly under the effect of PC1. Besides, the HSD groups kept a high degree of similarity between intra-group samples. In contrast, the BLSD group held a relative higher variation among samples. There was still a high degree of separation between the BLSD and LSD groups under the combined effect of PC1 and PC2. Venn diagram revealed that feeding time and diets may have a combined impact on fecal microbiota (**Figure 2B**). The number of shared OTUs between the BLSD and the LSD groups was 418, which was greater than those between the BHSD and the HSD groups (261). The numbers of OTUs specific for the four groups (BLSD, LSD, BHSD, and HSD) were 19, 44, 7, and 2, respectively. Compared to the LSD groups, the HSD groups had relatively lower diversity of OTUs. In other words, the high-salt diet might cause the reduction in gut microbial diversity.

**FIGURE 3:**
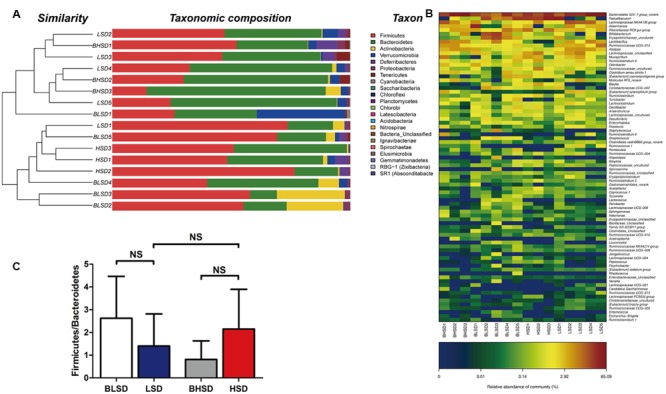
Clustering of gut microbiota. **(A)** Hierarchical clustering of fecal microbiota on the phylum level. Each line and bar represents the same sample. **(B)** Changes in the ratio of phyla Firmicutes/Bacteroidetes on the phylum level. The bars represent the standard deviations. Data were analyzed by one-way analysis of variance and means were compared by two sample’s *t*-test. **(C)** Heatmap of gut microbiota on the genus level. Each column represents one sample and each row represents one genus. Note: The numbers of animals for high-salt and low-salt diet groups are 3 and 5, respectively. BLSD, low-salt diet group before feeding; LSD, low-salt diet group after feeding; BHSD, high-salt diet group before feeding; HSD, high-salt diet group after feeding.

Hierarchical clustering analysis of fecal bacteria on the OTU level declared that the low-salt diet group showed a great intra-group variation among samples (**Figure 3A**). This was in agreement with the PCA results. On the phylum level, all fecal samples shared the similar community structure. Bacteroidetes and Firmicutes were the dominant phyla in all groups, regardless of diet and feeding time effects, contributing to 11.31∼75.23 and 14.42∼76.50% of the total OTUs, respectively. In the BHSD group, Bacteroidetes was the most abundant (55.27%), and followed by Firmicutes (32.20%), Verrucomicrobia (3.46%), Actinobacteria (2.45%), and Proteobacteria (1.32%). After feeding for 8 weeks (HSD group), the predominant phyla were changed. The dominant bacteria were Firmicutes (58.62%), and followed by Bacteroidetes (35.35%), Verrucomicrobia (1.17%), Actinobacteria (1.02%), and Proteobacteria (0.77%). In the BLSD group, Firmicutes was the predominant phyla (49.62%), and followed by Bacteroidetes (26.25%), Actinobacteria (12.96%), Proteobacteria (1.29%), and Verrucomicrobia (1.29%). After 8-week feeding (LSD group), the abundance of Firmicutes was decreased to 44.79%, while the abundance of Bacteroidetes was increased to 46.16%. The high-salt diet induced an increase in the ratio of Firmicutes to Bacteroidetes, while the low-salt diet tended to decrease the ratio (*P* > 0.05; **Figure 3B**).

On the genus level (**Figure 3C**), the differential sequences were matched with 50 genera. Bacteroidales S24-7 group was a predominant genus in each group, which had the highest abundance (45.40%), and was followed by Lachnospiraceae NK4A136 group (8.47%), *Lactobacillus* (6.95%), *Alistipes* (4.76%), Ruminococcaceae UCG-014 (4.20%), and *Akkermansia* (3.46%). After feeding for 8 weeks, *Faecalibaculum* became the predominant genus (30.18%), and followed by Bacteroidales S24-7 group (26.86%), Rikenellaceae RC9 gut group (7.31%), Lachnospiraceae (4.86%), Ruminococcaceae (3.79%), and Lachnospiraceae NK4A136 group (3.66%). In the BLSD group, Bacteroidales S24-7 group was the most abundant (18.84%), and followed by *Faecalibaculum* (12.90%), *Bifidobacterium* (9.62%), Erysipelotrichaceae (9.14%), *Akkermansia* (8.46%) and Rikenellaceae RC9 gut group (4.28%). After 8-week feeding, Bacteroidales S24-7 group was still the most abundant (37.81%), and followed by Lachnospiraceae NK4A136 group (9.76%), *Faecalibaculum* (9.61%), Ruminococcaceae UCG-014 (4.65%), *Alistipes* (4.22%), and *Lactobacillus* (3.61%).

#### Linear Discriminant Analysis of Fecal Microbiota

**FIGURE 4:**
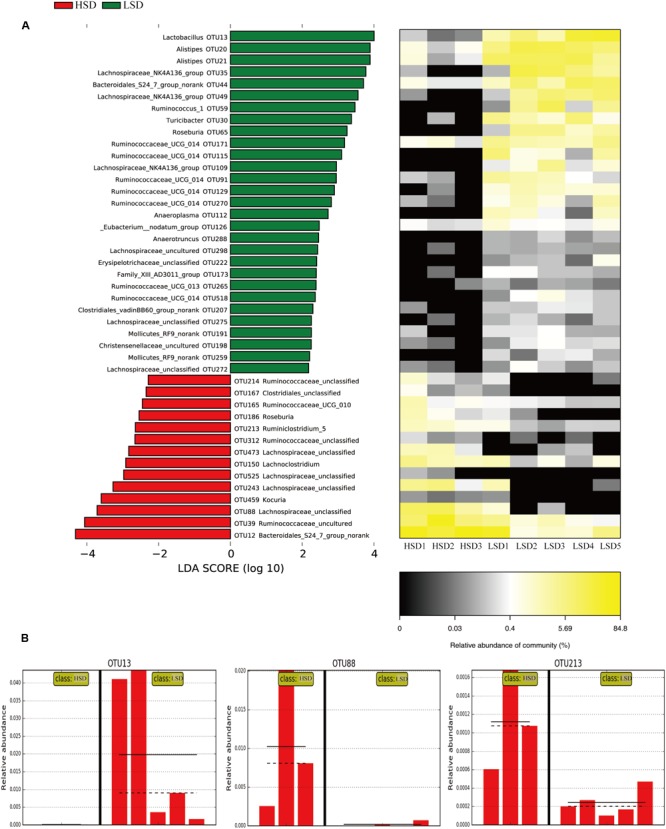
LEfSe analyses of gut microbiota data. **(A)** Comparisons of fecal microbiota by using LEfSe analysis. Bacterial OTUs rich in the low-salt diet group (green bars) and the high-salt diet group (red bars). Comparisons between the two groups are significant (*P* < 0.05, one-way ANOVA). Note: The left histogram shows the LDA scores calculated for characteristics at the OTU level, while the right heatmap shows the relative abundance of OTUs. Each column represents one sample and each row represents the OTU corresponding to the left one. **(B)** Relative abundances of *Lactobacillus*, Lachnospiraceae, and Ruminococcaceae in two diet groups. The means and medians are shown as solid and dashed lines in each group. Note: Each column represents one sample. HSD, high-salt diet group after feeding; LSD, low-salt diet group after feeding.

The above results showed that all samples can be divided into the HSD and the LSD classes. Each class had a similar composition of fecal microbiota. To identify microbial candidates that were specific for the two classes, LEfSe was adopted to determine particular biomarker. LEfSe analysis revealed 43 different OTUs between HSD and LSD classes (**Figure 4A**). Fourteen of these OTUs were higher in the HSD group and 29 OTUs were higher in the LSD group (*P* < 0.05). As compared to the LSD group, the HSD group had higher abundances (*P* < 0.05) of Lachnospiraceae (OTU473, OTU525, OTU243, and OTU88) and Ruminococcaceae (OTU214, OTU165, OTU213, and OTU312), but lower abundance (*P* < 0.05) of *Lactobacillus* (OTU13). Bacteroidales S24-7 (OTU12) was one of the most predominant bacteria in the HSD class, but its abundance was much lower in the LSD class (4.86 vs. 0.79%, *P* < 0.05). A butyrate-producing bacterium, *Roseburia* (OTU186) showed higher abundance in the HSD group. In contrast, the abundances of Lachnospiraceae NK4A136 (OTU49), Ruminococcaceae UCG-014 (OTU129 and OTU171), and *Alistipes* (OTU21 and OTU20) were higher in the LSD group. As shown above, genera *Lactobacillus*, Lachnospiraceae, and Ruminococcaceae were the specific bacteria. Therefore, three typical OTUs (OTU13, OTU88, and OTU213) were picked out for comparisons between diet groups (**Figure 4B**). These OTUs were significantly different between the low-salt and the high-salt diet groups. A multiple comparison indicated that mice fed high-salt diet had the higher abundance of Lachnospiraceae and Ruminococcaceae, but it was lower for the LSD group. Besides, the mice fed low-salt diet showed a higher abundance of *Lactobacillus* than the HSD group.

**FIGURE 5:**
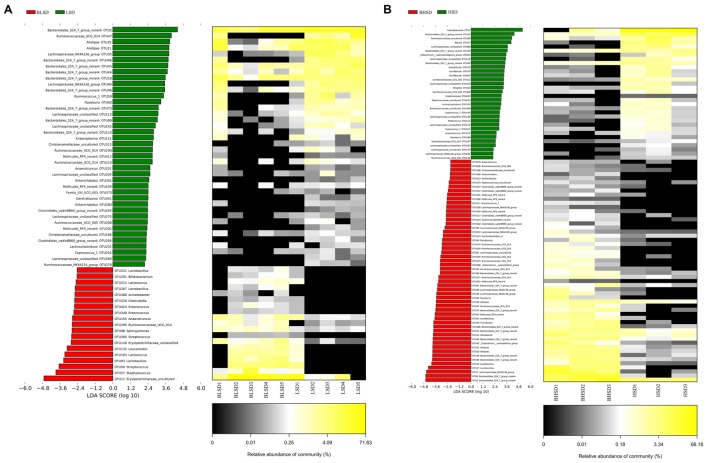
Changes of fecal microbiota at two time points by LEfSe analysis. **(A)** Between BLSD and LSD group. **(B)** Between BHSD and HSD group. Bacterial OTUs are rich in low-salt diet group before feeding (red bars) and after feeding (green bars). Comparisons between the two groups are significant (*P* < 0.05, one-way ANOVA). Note: The left histogram shows the LDA scores calculated for characteristics on the OTU level, while the right heatmap shows the relative abundances of OTUs. Each column represents one sample and each row represents the OTU corresponding to left one. BLSD, low-salt diet group before feeding; LSD, low-salt diet group after feeding. BHSD, high-salt diet group before feeding; HSD, high-salt diet group after feeding.

To evaluate time effect on the composition of fecal microbiota, LEfSe analysis was performed to distinguish bacterial taxa between the LSD groups before and after feeding (**Figure 5A**). The abundances of 59 OTUs were significantly different between BLSD and LSD groups. Nineteen OTUs were more abundant in the BLSD group than those of the LSD group, including Lactobacillales (OTU521, OTU212, OTU267, OTU103, and OTU93), Ruminococcaceae UCG_014 (OTU295), *Leuconostoc mesenteroides* (OTU132), *Streptococcus* (OTU58), and Erysipelotrichaceae (OTU118 and OTU11). This indicated that these bacteria showed a negative response to the low-salt diet. On the other hand, the abundances of the other 40 OTUs had a positive response to the low-salt diet, including Bacteroidales S24-7 (OTU2, OTU18, OTU44, OTU45, OTU46, OTU73, OTU89, OTU110, and OTU346,), Ruminococcaceae (OTU47, OTU59, OTU190, OTU206, OTU215, and OTU276), *Alistipes* (OTU20 and OTU21), and Lachnospiraceae OTUs (OTU35, OTU49, OTU113, OTU143, OTU204, OTU275, and OTU269). In addition, Mollicutes RF9 (OTU239), *Roseburia* (OTU65), and *Anaeroplasma* (OTU112) were also higher in abundance in the LSD group.

In the high-salt diet groups, there were 56 OTUs whose abundances were significantly changed (**Figure 5B**). The abundances of 32 OTUs were increased after intake of high-salt diet, while the abundances of the other 24 OTUs were decreased (*P* < 0.05). The increased OTUs belong to Lachnospiraceae_NK4A136 (OTU109, OTU99, OTU533, OTU49, OTU35, and OTU7), Lachnospiraceae (OTU88, OTU123, OTU243, OTU138, OTU473, OTU192, and OTU179), and *Oscillibacter* (OTU78 and OTU60). The decreased OTUs belong to Bacteroidales S24-7 (OTU61, OTU73, OTU346, OTU23, OTU45, OTU19, OTU44, OTU6, and OTU2), *Alistipes* (OTU38, OTU21, and OTU20), Ruminococcaceae UCG-014 (OTU270, OTU518, OTU129, OTU115, OTU91, OTU171, and OTU47), and *Lactobacillus* (OTU53).

### High-Salt Diet Affected Intestinal Proteome Profiles

To determine the effect of diets on protein expression in mice, proteomic analysis was carried out on intestinal contents of mice. Data were matched against diet, host, and gut microbiota database, and produced several qualitative and quantification results.

#### Dietary Proteins

**FIGURE 6:**
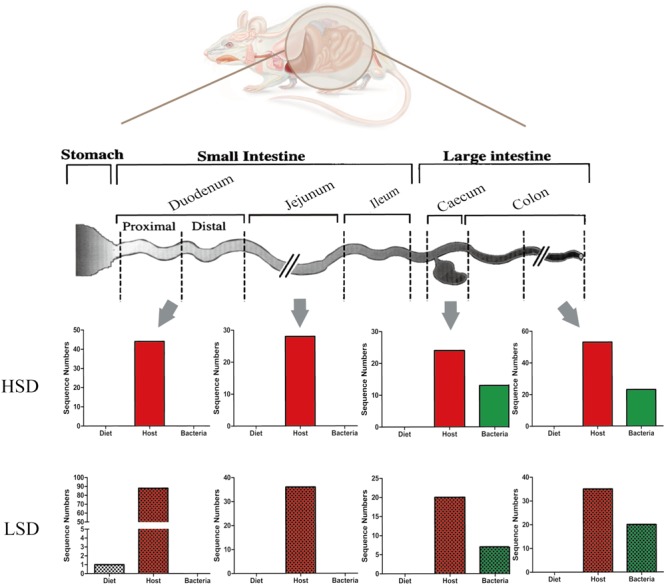
Changes of peptide numbers in the intestinal tract of mice. The intestinal contents came from duodenum, jejunum, cecum, and colon. Bars with mosaic represent LSD group, and those with pure color represent HSD group.

A total of 452 protein groups were identified from all gut contents by searching against the *Bos taurus* (bovine) database. After filtration, 54 proteins were matched. The majority of them were casein kinase and other enzymes. Only one peptide was matched with β-lactoglobulin (fragment) in the LSD group (**Figure 6**). No casein fragments were detected in the HSD group. The results indicated that casein can be easily degraded and absorbed in the digestive tract, and thus there was no significant difference in the composition of diet-based protein fragments between the LSD and the HSD groups. The abundance of diet-based protein fragments decreased from the upper to the lower gastrointestinal tract. Therefore, results suggested that casein was susceptible to peptic and tryptic digestion and absorption in gastrointestinal tract.

#### Host Proteins

In the same samples, 179 and 149 unique peptides were identified and matched with the database in the LSD and the HSD groups. A total of 20 unique proteins were differentially expressed in duodenum contents, both had lower abundance in the HSD group (Supplementary Table S2). The numbers of host-based peptides appeared to decrease from the duodenal to the cecal contents, and then increase from the cecal to colonic contents (**Figure 6**). Several differential proteins were digestive enzymes, including chymotrypsinogen B precursor, pancreatic triacylglycerol lipase precursor, carboxypeptidase A1 precursor, and carboxypeptidase B1, which had lower abundance in HSD group as compared to the LSD group (*P* < 0.05). These results indicated that high-salt diet may inhibit the excretion of the digestive enzymes. In addition, several cytoplasmic components, including alpha-actin, tropomyosin alpha-1 chain, cadherin 17, and vesicle membrane protein VAT-1 homolog were identified and their abundances were observed significantly different between the HSD and the LSD groups (*P* < 0.05). Of them, cadherin 17 is related to calcium ion binding, and vesicle membrane protein VAT-1 homolog is correlated with oxidoreductase activity, while phosphoglycerate mutase 1 involves glycolysis.

**FIGURE 7:**
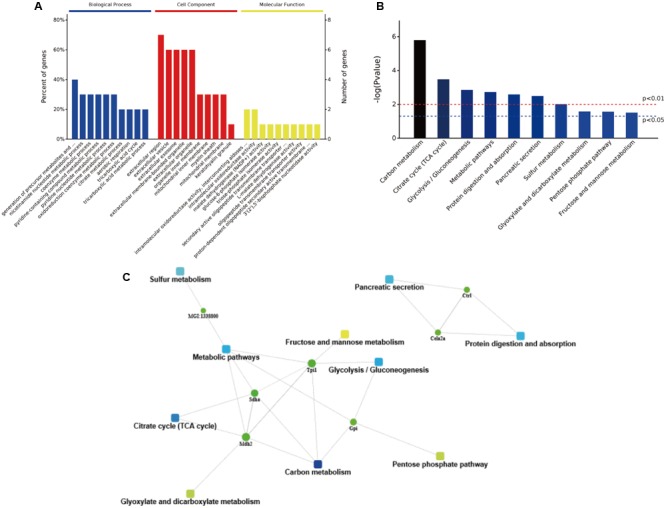
The bioinformatics analysis of differentially expressed proteins. **(A)** The GO annotation of identified NP-related proteins in three categories: biological process, cellular composition, and molecular function. **(B)** The distribution of enriched KEGG pathway of differentially expressed proteins. **(C)** A protein–protein interaction networks of identified differentially expressed proteins in duodenum contents. Different clusters of interacting proteins were identified using STRING.

The obtained protein data were analyzed using bioinformatics tools, in order to extract information relevant to involved pathways. The GO analysis indicated that in the biological process analysis, the majority of identified proteins were assigned to metabolic processes, especially in pyridine and coenzyme metabolic process. The cell component analysis declared that most of identified proteins belong to organelle and extracellular components. In the molecular functional analysis, most of the identified proteins were involved in enzyme activities, including oxidoreductase, malate dehydrogenase, glucose-6-phosphate isomerase, and triose-phosphate isomerase (**Figure 7A**). Then, the KEGG analysis indicated that active pathways involved were those related to secretion and metabolism, including protein digestion, absorption, and pancreatic secretion (**Figure 7B**). The results suggested that the high-salt diet may have an inhibitory effect on pancreatic secretion, by inhibiting the secretion of trypsin, thus leading to lower protein digestion and absorption efficiency compared to the LSD groups. In order to further explore more relevant information from the identified proteins, a more comprehensive bioinformatics analysis (STRING) of the protein data was performed to integrate protein–protein interaction networks. Seven proteins were recognized as key nodes in biological interaction networks (**Figure 7C**). Chymotrypsin-like elastase family member 2A precursor (P05208) and Chymopasin (Q9E205) were highly correlated with pancreatic secretion, protein digestion, and absorption. Proteins coded by Sdha and Tpi1 were involved in many metabolism processes, including succinate metabolic process, gluconeogenesis, and pentose-phosphate shunt process.

#### Bacterial Proteins

A total of 63 peptides were unambiguously identified and quantified, of which 18 proteins were uncharacterized. A variety of proteins and polysaccharides degrading enzymes were secreted by gut microbiota. In colon contents, 17 microbial proteins were common in the low- and the high-salt diet groups. Five proteins had a higher abundance in the high-salt diet group than those in the LSD group (Supplementary Table S3), including cytidylate kinase, trigger factor, 6-phosphogluconate dehydrogenase, transporter, and undecaprenyl-diphosphatase, which were secreted by different gut bacteria, including *Atopobium parvulum, Anaerococcus prevotii, Lactobacillus brevis, Anaerostipes hadrus*, and *Streptococcus agalactiae*. Cytidylate kinase is involved in pyrimidine metabolism, ATP binding, transferase, and cytidylate kinase activity. Trigger factor is in connection with protein transport, which acts as a chaperone by keeping the newly synthesized secretory and non-secretory proteins. Besides, trigger factor is involved in cell cycle, cell division, protein folding, and peptidyl-prolyl *cis–trans* isomerase activity, thus regulating its activity. The 6-phosphogluconate dehydrogenase was involved in the pentose phosphate pathway. Transport protein is a protein that ensures the function of transferring materials within an organism, which acts a crucial role as sodium symporter. Therefore, high concentrations of sodium may lead to comparable high expressions of transport protein. Undecaprenyl-diphosphatase was involved in dephosphorylation, peptidoglycan biosynthetic, and regulation of cell shape. Two proteins (acetylglutamate kinase and PBSX phage manganese-containing catalase) had relatively higher abundance in LSD group, which were secreted by *Clostridium beijerinckii* and *Bacillus subtilis* BEST7613. Acetylglutamate kinase is involved in arginine and proline biosynthetic and ATP-binding, which catalyzes the ATP-dependent phosphorylation of *N*-acetyl-glutamate. PBSX phage manganese-containing catalase is produced by *B. subtilis*, the intracellular catalase activity was decreased when cells were in anaerobic environment, while under aerobic conditions, an increasing trend will be noted with the removal of thiosulfate from the medium, or addition of manganese.

## Discussion

A high-salt diet has been shown to increase the risk to several diseases in children and adults (He et al., 2013). Previous studies focused on the cardiovascular disease and immune disease in response to high-salt diet. A high salt intake during pregnancy modified protein expression in offspring kidneys and reduced the final counts of glomeruli, thus increasing the risk of hypertension later in life (Koleganova et al., 2011). A diet rich in salt would increase the incidence of cardiovascular disease and blood pressure, after long-time feeding (Baldo et al., 2015). However, clinical data suggested that a high salt intake may increase the morbidity of numerous adverse effects (kidney damage, gastric cancer) that are independent of effects on blood pressure (Turlova and Feng, 2013). The present study provides some evidence that high-salt diet may affect body health by altering digestion and absorption of dietary proteins, secretion of host enzymes, and gut microbiota composition.

Food digestion and absorption is a dynamic process because dietary proteins are degraded and absorbed in the small intestine where the host excretes quite a lot of digestive enzymes, and some dying epithelial cells may be separated into the lumen (Wang et al., 2016). Diet- and host-based proteins would enter into the cecum and colon, in which microbial fermentation utilizes the majority of such proteins. On the other hand, gut microbiota proteins are dominant in the colon. In total, the level of protein kept in balance between the cecum and the colon. And thus, the feasibility of traditional method for the diet protein bioavailability (Stein et al., 2007) needs to be re-evaluated. In the present study, casein is the sole protein in diet formulation which has been known to have high bioavailability. Very few fragments of casein can be identified in the duodenal contents and none of casein fragments were detectable in the jejunal, cecal, and colonic contents, indicating that casein can be easily digested by digestive enzymes into much smaller pieces that were not detectable by the LC-MS/MS methods. High-salt diet seemed not affect the digestion of dietary casein, but it has been reported to reduce the abundances of 20 proteins involved in carbohydrate, amino acid, and energy metabolism as compared to low-salt diet (Mayyas et al., 2017). It is well known that food digestion and absorption mainly occurs in the small intestine, especially for duodenum and jejunum. Therefore, many digestive enzymes were secreted into duodenum to complete digestion. In the present study, the differentially expressed proteins were mainly components or precursors of digestive enzymes in duodenal contents, including enzymes associated with proteolysis (chymotrypsinogen B precursor, carboxypeptidase A1 precursor, carboxypeptidase B1, chymotrypsin-like elastase family member 2A precursor, and chymopasin) and enzymes associated with fat decomposition (pancreatic triacylglycerol lipase precursor). It was suggested that the effects of high-salt diet were associated with reduced degradation of proteins, which remained in the intestine for a short time than usual. Although sodium chloride needs not to be digested, sodium and chloride ions would help us to digest and absorb the nutrients, the presence of sodium is still critical when the intestinal cells move nutrients into the blood stream.

A high-salt diet was also observed to have a certain effect on gut microbiota composition and microbial proteins in gut contents. Recent studies indicated that the HSD alone induced changes in microbiota profiles, characterized by a loss in diversity and a shift in its composition. The present study further confirmed such a diet effect by using high-throughput sequencing. The Bacteroidetes, Firmicutes, Actinobacteria, Proteobacteria, and Verrucomicrobia were predominant phyla in fecal samples, constituting the core microbiome of mice. Mice fed the high-salt diet had higher Firmicutes but lower Bacteroidetes than those fed the low-salt diet, resulting in an increase in the Firmicutes/Bacteroidetes ratio that is associated with cardiovascular diseases and obesity (Bäckhed et al., 2004; Ley et al., 2006; Jose and Raj, 2015). Additionally, the HSD group had higher abundances of Lachnospiraceae and *Ruminococcus* OTUs but lower abundance of *Lactobacillus* OTU as compared to the LSD group, which was in line with previous studies (Miranda et al., 2016). Sodium chloride absorption appeared to be mediated by a process of bulk flow of solution along osmotic pressure gradient (Fordtran et al., 1968). As one member of the SLC9A family expressed in the gut, epithelial Na^+^/H^+^ exchange is an effective membrane transport mechanism that is engaged in intestinal sodium chloride transport, in the regulation of the extracellular milieu to promote nutrients absorption, and to regulate the gut microbial environment. Besides, Na^+^/H^+^ exchange was a vital process for maintaining microbial homeostasis within the gut (Kiela et al., 2006). Studies have shown that high sodium intake would increase the activity of apical Na^+^/H^+^ exchange by 30%, which basolateral Na^+^/H^+^ exchange was inhibited but did not change NHE3 abundance (Good et al., 2011). Clinical data suggested that perturbations in intestinal Na^+^/H^+^ exchange located at the apical or basolateral membranes have undertaken more basal functions as nutrient or electrolyte transporters to modulate epithelial barrier function and gut microbiota (Gurney et al., 2017). Intestinal NHE3 expression and function was inhibited by enteropathogenic bacteria (*Escherichia coli* and *Clostridium difficile*) (Hecht et al., 2004; Larmonier et al., 2013; Engevik et al., 2015), whereas, upregulated by commensal *Lactobacillus acidophilus* (Singh et al., 2012). Therefore, the mechanism of high-salt diet shaping gut microbiota may be as follows: high sodium intake may increase the activity of Na^+^/H^+^ exchange which is highly correlated with gut microbiota, and then lead to the alterations of microbial profiles. It is difficult for us to check how high-salt diet affects the composition of gut microbiota. This is because high-salt diet may induce mice to drinking more water and excreting more urine to remove excessive salt. Besides, high sodium intake does not cause total body water storage but induce a relative fluid shift from the interstitial into the intravascular space (Heer et al., 2000). And thus we postulate that high-salt diet may affect the excretion of digestive enzymes in the small intestine that enter into the large intestine and further affect gut microbiota composition.

## Conclusion

Intake of high-salt diet did not cause dramatic variations in feed intake, weight gain, and the levels of several inflammatory factors. However, a high-salt diet may inhibit the excretion of digestive enzymes from the host, change biological process, cell component, and molecular function in duodenal contents, and further alter the gut microbiota composition, leading to higher ratio of Firmicutes to Bacteroidetes, higher abundance of Lachnospiraceae and *Ruminococcus* but lower abundance of *Lactobacillus*.

## Author Contributions

The eight authors are justifiably credited with authorship, according to the authorship criteria. In detail: CL and CW designed the research; CW and CL wrote the manuscript; CW, ZH, KYu, KYe, and RD acquired all raw data; GZ and XX analyzed data analysis and criticized the manuscript. All authors reviewed the manuscript.

## Conflict of Interest Statement

The authors declare that the research was conducted in the absence of any commercial or financial relationships that could be construed as a potential conflict of interest.

## Footnotes

1 http://qiime.org/

2 http://drive5.com/uparse/

3 http://rdp.cme.msu.edu/

4 http://bioinformatics.org.au/tools/hclust/

5 http://huttenhower.sph.harvard.edu/galaxy/

6 www.uniprot.org

7 http://pir.georgetown.edu/

8 www.omicsbean.cn

## Supplementary Material

FIGURE S1 Rarefaction curves of fecal microbiota in all samples. Note: Each line represents one sample

## References

[B1] Amato K. R. Yeoman C. J. Kent A. Righini N. Carbonero F. Estrada A. (2013). Habitat degradation impacts black howler monkey (*Alouatta pigra*) gastrointestinal microbiomes. *ISME J.* 7 1344–1353. 10.1038/ismej.2013.16 23486247 PMC3695285

[B2] Bäckhed F. Ding H. Wang T. Hooper L. V. Koh G. Y. Nagy A. (2004). The gut microbiota as an environmental factor that regulates fat storage. *Proc. Natl. Acad. Sci. U.S.A.* 101 15718–15723. 10.1073/pnas.0407076101 15505215 PMC524219

[B3] Baldo M. P. Rodrigues S. L. Mill J. G. (2015). High salt intake as a multifaceted cardiovascular disease: new support from cellular and molecular evidence. *Heart Fail. Rev.* 20 461–474. 10.1007/s10741-015-9478-7 25725616

[B4] Clemente J. C. Ursell L. K. Parfrey L. W. Knight R. (2012). The impact of the gut microbiota on human health: an integrative view. *Cell* 148 1258–1270. 10.1016/j.cell.2012.01.035 22424233 PMC5050011

[B5] Culligan E. P. Marchesi J. R. Hill C. Sleator R. D. (2014). Combined metagenomic and phenomic approaches identify a novel salt tolerance gene from the human gut microbiome. *Front. Microbiol.* 5:189 10.3389/fmicb.2014.00189 PMC4010731 24808895

[B6] Daniel H. Gholami A. M. Berry D. Desmarchelier C. Hahne H. Loh G. (2014). High-fat dietalters gut microbiota physiology in mice. *ISME J.* 8 295–308. 10.1038/ismej.2013.155 24030595 PMC3906816

[B7] Engevik M. A. Engevik K. A. Yacyshyn M. B. Wang J. Hassett D. J. Darien B. (2015). Human *Clostridium difficile* infection: inhibition of NHE3 and microbiota profile. *Am. J. Physiol. Gastrointest. Liver Phyiol.* 308 G497–G509. 10.1152/ajpgi.00090.2014 PMC4422371 25552580

[B8] Fordtran J. S. Rector F. C. Jr. Carter N. W. (1968). The mechanisms of sodium absorption in the human small intestine. *J. Clin. Invest.* 47 884–900. 10.1172/JCI105781 5641624 PMC297237

[B9] Good D. W. George T. Watts B. A. (2011). High sodium intake increases HCO3^-^ absorption in medullary thick ascending limb through adaptations in basolateral and apical Na^+^/H^+^ exchangers. *Am. J. Physiol. Renal Physiol.* 301 F334–F343. 10.1152/ajprenal.00106.2011 21613418 PMC3154595

[B10] Gurney M. A. Laubitz D. Ghishan F. K. Kiela P. R. (2017). Pathophysiology of intestinal Na^+^/H^+^ exchange. *Cell. Mol. Gastroenterol. Hepatol.* 3 27–40. 10.1016/j.jcmgh.2016.09.010 28090568 PMC5235326

[B11] Hauck S. M. Dietter J. Kramer R. L. Hofmaier F. Zipplies J. K. Amann B. (2010). Deciphering membrane-associated molecular processes in target tissue of autoimmune uveitis by label-free quantitative mass spectrometry. *Mol. Cell. Proteomics* 9 2292–2305. 10.1074/mcp.M110.001073 20601722 PMC2953921

[B12] He F. J. Li J. Macgregor G. A. (2013). Effect of longer term modest salt reduction on blood pressure: Cochrane systematic review and meta-analysis of randomised trials. *BMJ* 346:f1325 10.1136/bmj.f1325 23558162

[B13] He F. J. MacGregor G. A. (2009). A comprehensive review on salt and health and current experience of worldwide salt reduction programmes. *.J. Hum. Hypertens.* 23 363–384. 10.1038/jhh.2008.144 19110538

[B14] Hecht G. Hodges K. Gill R. K. Kear F. Tyagi S. Malakooti J. (2004). Differential regulation of Na^+^/H^+^ exchange isoform activities by enteropathogenic *E. coli* in human intestinal epithelial cells. *Am. J. Physiol. Gastrointest. Liver Phyiol.* 287 G370–G378. 10.1152/ajpgi.00432.2003 15075254

[B15] Heer M. Baisch F. Kropp J. Gerzer R. Drummer C. (2000). High dietary sodium chloride consumption may not induce body fluid retention in humans. *Am. J. Physiol. Renal Physiol.* 278 F585–F595. 10751219 10.1152/ajprenal.2000.278.4.F585

[B16] Jose P. A. Raj D. (2015). Gut microbiota in hypertension. *Curr. Opin. Nephrol. Hypertens.* 24 403–409. 10.1097/MNH.0000000000000149 26125644 PMC4578629

[B17] Kiela P. R. Xu H. Ghishan F. K. (2006). Apical NA^+^/H^+^ exchangers in the mammalian gastrointestinal tract. *J. Physiol. Pharmacol.* 57 51–79. 17228096

[B18] Koleganova N. Piecha G. Ritz E. Becker L. E. Müller A. Weckbach M. (2011). Both high and low maternal salt intake in pregnancy alter kidney development in the offspring. *Am. J. Physiol. Renal Physiol.* 301 F344–F354. 10.1152/ajprenal.00626.2010 21593188

[B19] Larmonier C. B. Laubitz D. Hill F. M. Shehab K. W. Lipinski L. Midura-Kiela M. T. (2013). Reduced colonic microbial diversity is associated with colitis in NHE3-deficient mice. *Am. J. Physiol. Gastrointest. Liver Physiol.* 305 G667–G677. 10.1152/ajpgi.00189.2013 24029465 PMC3840234

[B20] Ley R. E. Turnbaugh P. J. Klein S. Gordon J. I. (2006). Microbial ecology: human gut microbes associated with obesity. *Nature* 444 1022–1023. 10.1038/4441022a 17183309

[B21] Lozupone C. Lladser M. E. Knights D. Stombaugh J. Knight R. (2011). UniFrac: an effective distance metric for microbial community comparison. *ISME J.* 5 169–172. 10.1038/ismej.2010.133 20827291 PMC3105689

[B22] Mayyas F. Alzoubi K. H. Al-Taleb Z. (2017). Impact of high fat/high-salt diet on myocardial oxidative stress. *Clin. Exp. Hypertens.* 39 126–132. 10.1080/10641963.2016.1226894 28287889

[B23] Meneton P. Jeunemaitre X. de Wardener H. E. MacGregor G. A. (2005). Links between dietary salt intake, renal salt handling, blood pressure, and cardiovascular diseases. *Physiol. Rev.* 85 679–715. 10.1152/physrev.00056.2003 15788708

[B24] Micha R. Michas G. Mozaffarian D. (2012). Unprocessed red and processed meats and risk of coronary artery disease and type 2 diabetes–an updated review of the evidence. *Curr. Atheroscler. Rep.* 14 512–524. 10.1007/s11883-012-0282-8 PMC3483430 23001745

[B25] Millen B. E. Abrams S. Adams-Campbell L. Anderson C. A. Brenna J. T. Campbell W. W. (2016). The 2015 dietary guidelines advisory committee scientific report: development and major conclusion. *Adv. Nutr.* 7 438–444. 10.3945/an.116.012120 27184271 PMC4863277

[B26] Miranda P. M. Serkis V. de Palma G. Pigrau M. Lu J. Collins S. (2016). Su1901 high-salt diet increases susceptibility to experimental colitis: a putative role of gut microbiota. *Gastroenterology* 150 S583 10.1016/S0016-5085(16)32000-5

[B27] Mugavero K. L. Gunn J. P. Dunet D. O. Bowman B. A. (2014). Sodium reduction: an important public health strategy for heart health. *J. Public Health Manag. Pract.* 20 S1–S5. 10.1097/PHH.0b013e3182aa659c PMC4450095 24322810

[B28] Obih P. O. Oyekan A. (2014). Proteomic analysis of salt-induced changes in protein expression in PPAR [alpha] null mice. *Pharmacol. Pharm.* 5 996–1005. 10.4236/pp.2014.511111

[B29] Park J. Y. Seong J. K. Paik Y. K. (2004). Proteomic analysis of diet-induced hypercholesterolemic mice. *Proteomics* 4 514–523. 10.1002/pmic.200300623 14760724

[B30] Putignani L. Del Chierico F. Petrucca A. Vernocchi P. Dallapiccola B. (2014). The human gut microbiota: a dynamic interplay with the host from birth to senescence settled during childhood. *Pediatr. Res.* 76 2–10. 10.1038/pr.2014.49 24732106

[B31] Rist V. T. Weiss E. Eklund M. Mosenthin R. (2013). Impact of dietary protein on microbiota composition and activity in the gastrointestinal tract of piglets in relation to gut health: a review. *Animal* 7 1067–1078. 10.1017/S1751731113000062 23410993

[B32] Schloss P. D. Westcott S. L. Ryabin T. Hall J. R. Hartmann M. Hollister E. B. (2009). Introducing mothur: open-source, platform-independent, community-supported software for describing and comparing microbial communities. *Appl. Environ. Microbiol.* 75 7537–7541. 10.1128/AEM.01541-09 19801464 PMC2786419

[B33] Segata N. Izard J. Waldron L. Gevers D. Miropolsky L. Garrett W. S. (2011). Metagenomic biomarker discovery and explanation. *Genome Biol.* 12:R60 10.1186/gb-2011-12-6-r60 PMC3218848 21702898

[B34] Shi X. Li C. Cao M. Xu X. Zhou G. Xiong Y. L. (2016). Comparative proteomic analysis of longissimus dorsi muscle in immuno-and surgically castrated male pigs. *Food Chem.* 199 885–892. 10.1016/j.foodchem.2015.11.059 26776048

[B35] Singh V. Raheja G. Borthakur A. Kumar A. Gill R. K. Alakkam A. (2012). *Lactobacillus acidophilus* upregulates intestinal NHE3 expression and function. *Am. J. Physiol. Gastrointest. Liver Phyiol.* 303 G1393–G1401. 10.1152/ajpgi.00345.2012 PMC3532544 23086913

[B36] Stein H. H. Sève B. Fuller M. F. Moughan P. J. de Lange C. F. Committee on Terminology to Report AA Bioavailability and Digestibility (2007). Invited review: amino acid bioavailability and digestibility in pig feed ingredients: terminology and application. *J. Anim. Sci.* 85 172–180. 10.2527/jas.2005-742 17179553

[B37] Strazzullo P. D’Elia L. Kandala N. B. Cappuccio F. P. (2009). Salt intake, stroke, and cardiovascular disease: meta-analysis of prospective studies. *BMJ* 339:b4567 10.1136/bmj.b4567 PMC2782060 19934192

[B38] Sun N. Sun W. Li S. Yang J. Yang L. Quan G. (2015). Proteomics analysis of cellular proteins co-immunoprecipitated with nucleoprotein of influenza a virus (H7N9). *Int. J. Mol. Sci.* 16 25982–25998. 10.3390/ijms161125934 26528969 PMC4661793

[B39] Trawńska B. Polonis A. Lechowski J. Tymczyna L. Borowski R. Gizińska K. (2013). Effect of the addition of magnesium salt to a feed mixture on intestinal microflora, health, and production of sows. *Bull. Vet. Inst. Pulawy* 57 69–72. 10.2478/bvip-2013-0013

[B40] Turlova E. Feng Z. P. (2013). Dietary salt intake and stroke. *Acta Pharmacol. Sin.* 34 8–9. 10.1038/aps.2012.179 23274412 PMC4086497

[B41] Wang C. Wang B. Li B. (2016). Bioavailability of peptides from casein hydrolysate in vitro: amino acid compositions of peptides affect the antioxidant efficacy and resistance to intestinal peptidases. *Food Res. Int.* 81 188–196. 10.1016/j.foodres.2015.12.013

[B42] Wen S. Zhou G. Song S. Xu X. Voglmeir J. Liu L. (2015). Discrimination of in vitro and in vivo digestion products of meat proteins from pork, beef, chicken, and fish. *Proteomics* 15 3688–3698. 10.1002/pmic.201500179 26227428 PMC5049642

[B43] Xiao F. Crissey M. A. Lynch J. P. Kaestner K. H. Silberg D. G. Suh E. (2005). Intestinal metaplasia with a high salt diet induces epithelial proliferation and alters cell composition in the gastric mucosa of mice. *Cancer Biol. Ther.* 4 669–675. 10.4161/cbt.4.6.1734 15970710

[B44] Yi B. Titze J. Rykova M. Feuerecker M. Vassilieva G. Nichiporuk I. (2015). Effects of dietary salt levels on monocytic cells and immune responses in healthy human subjects: a longitudinal study. *Transl. Res.* 166 103–110. 10.1016/j.trsl.2014.11.007 25497276 PMC5538905

[B45] Zhu Y. Lin X. Zhao F. Shi X. Li H. Li Y. (2015). Meat, dairy and plant proteins alter bacterial composition of rat gut bacteria. *Sci. Rep.* 5:15220 10.1038/srep15220 PMC4604471 26463271

