# Supplementary figures and images for: High-Salt Diet Has a Certain Impact on Protein Digestion and Gut Microbiota: A Sequencing and Proteome Combined Study
Source: Front Microbiol. 2017 Sep 21;8:1838. doi: 10.3389/fmicb.2017.01838 (PMC5627008; doi:10.3389/fmicb.2017.01838)

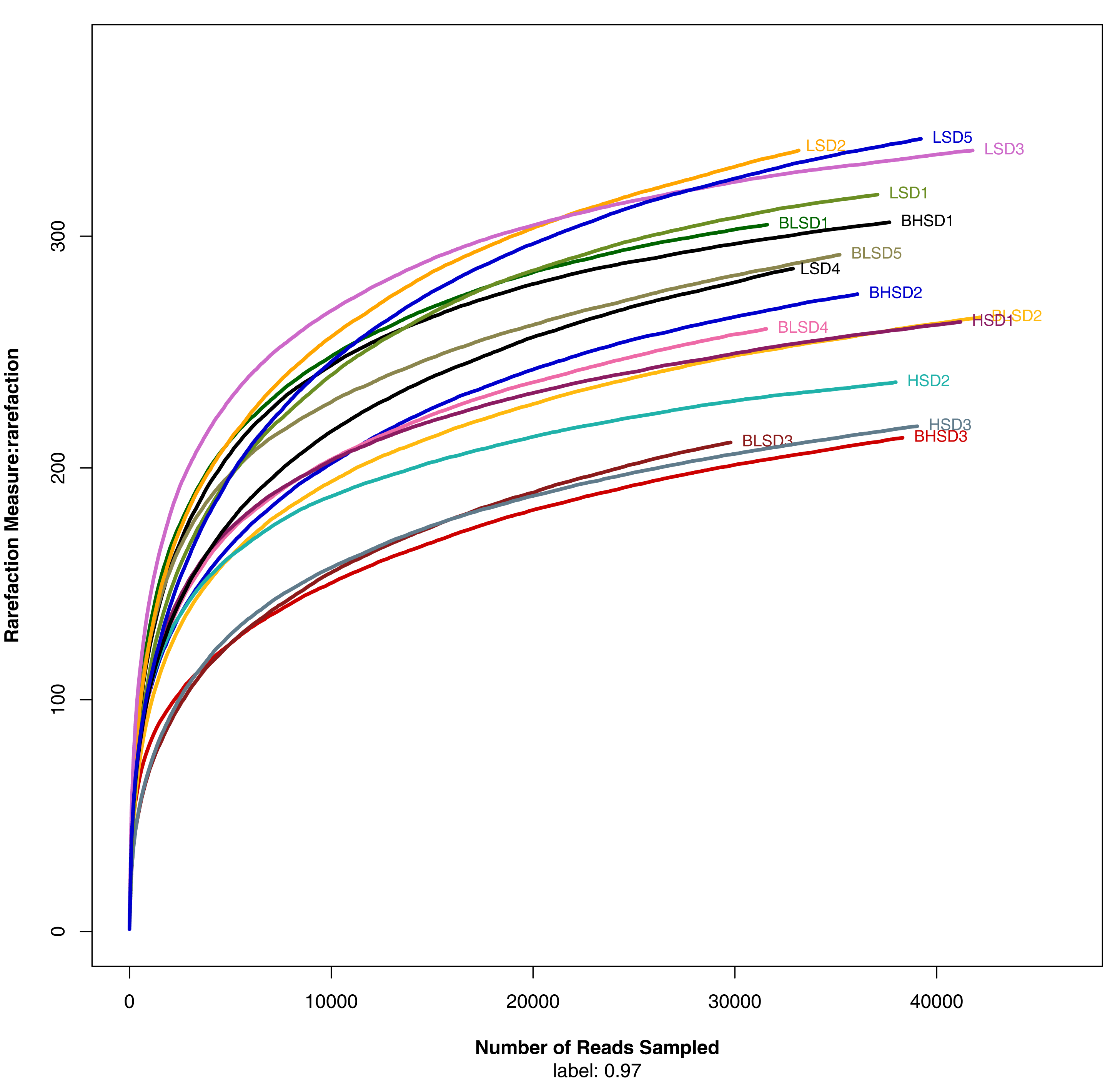

Supplement: FIGURE S1 — Rarefaction curves of fecal microbiota in all samples. Note: Each line represents one sample [file Image_1.TIF]
